# QADI as a New Method and Alternative to Kappa for Accuracy Assessment of Remote Sensing-Based Image Classification

**DOI:** 10.3390/s22124506

**Published:** 2022-06-14

**Authors:** Bakhtiar Feizizadeh, Sadrolah Darabi, Thomas Blaschke, Tobia Lakes

**Affiliations:** 1 Department of Remote Sensing and GIS, University of Tabriz, Tabriz 516661647, Iran; darabisadra@gmail.com; 2 GIScience Lab, Humboldt-Universität zu Berlin, 10117 Berlin, Germany; tobia.lakes@geo.hu-berlin.de; 3 Department of Geoinformatics-Z-GIS, University of Salzburg, 5020 Salzburg, Austria; thomas.blaschke@sbg.ac.at; 4 IRI THESys, Humboldt-Universität zu Berlin, 10117 Berlin, Germany

**Keywords:** remote sensing, accuracy assessment, alternative to traditional Kappa, image classification

## Abstract

Classification is a very common image processing task. The accuracy of the classified map is typically assessed through a comparison with real-world situations or with available reference data to estimate the reliability of the classification results. Common accuracy assessment approaches are based on an error matrix and provide a measure for the overall accuracy. A frequently used index is the Kappa index. As the Kappa index has increasingly been criticized, various alternative measures have been investigated with minimal success in practice. In this article, we introduce a novel index that overcomes the limitations. Unlike Kappa, it is not sensitive to asymmetric distributions. The quantity and allocation disagreement index (QADI) index computes the degree of disagreement between the classification results and reference maps by counting wrongly labeled pixels as A and quantifying the difference in the pixel count for each class between the classified map and reference data as Q. These values are then used to determine a quantitative QADI index value, which indicates the value of disagreement and difference between a classification result and training data. It can also be used to generate a graph that indicates the degree to which each factor contributes to the disagreement. The efficiency of Kappa and QADI were compared in six use cases. The results indicate that the QADI index generates more reliable classification accuracy assessments than the traditional Kappa can do. We also developed a toolbox in a GIS software environment.

## 1. Introduction

Earth observation technology and remote sensing methods are critical for a variety of environmental applications. The range of satellite sensors and the volume of remote sensing data has increased, as has the user base and the variety of methods and methodologies to process large amounts of data [1,2]. In addition, the spatial, spectral, and temporal resolutions of satellite images have increased, and data have become more accessible. It is sometimes argued that progress in technology and data analysis and increasing demand for efficient and cost-effective data-driven approaches have revolutionized Earth Observation methods [2,3]. Recent work illustrates the demand from various application fields for effective data-driven solutions [4,5,6,7,8,9,10,11,12,13]. Several efficient data-driven approaches (e.g., semi/automated and machine learning methods, deep learning, conventional network, etc.) have been proposed and implemented in recent years [7,14,15].

At the same time, novel data-driven approaches demand efficient accuracy assessment methods [13]. However, despite significant progress in image classification techniques, the development of accuracy assessment methods has not kept pace. This may seem surprising since the accuracy assessment and validation of results are critical steps in the process of image classification [16]. The purpose of the accuracy assessment is to indicate the degree to which the results derived from image classification agree with reality or conform to the ‘truth’ [17]. Accuracy estimates are usually empirical estimates of map properties, and, as with all statistical estimates, they have uncertainties [18]. This encompasses the validation of thematic maps obtained by classifying remotely sensed imagery by expressing the degree of ‘correctness’ of a classified map [19]. Specifically, accuracy assessment methods are used to compute the difference between a classified map and reference data.

Several accuracy assessment methods have been proposed to assess remote sensing-based classification accuracy [17,20,21,22,23,24,25,26,27]. The considered criteria can be a function of variables, such as the characteristics of the remotely sensed satellite imagery (e.g., the spatial- and spectral resolution), details of the defined classes (e.g., number and details of classes), and user preferences (e.g., tolerance to error). Thus far, no universal guidelines have been proposed or developed for assessing the accuracy of thematic maps based on remote sensing image classification [17]. A significant challenge in accuracy assessment lies in determining the minimum level of accuracy required to validate the results. The answer to the question of what constitutes a “good” classification result remains. Remote sensing-based classified maps must be validated in reference to the magnitude of their estimated accuracy. According to Lillesand and Kiefer [16], the minimum level of accuracy for results of a remote sensing-based classified map to be considered valid is ≥85%, which the remote sensing community has widely accepted as a target in image classification.

In the context of the accuracy assessment, the error matrix, which is assigned based on ground control data, is the most common and widely applied method to validate classified maps in remote sensing [28]. The confusion/error matrix is a traditional accuracy assessment approach that cross-tabulates classified data against observed reference data. This cross-tabulation computation produces several measures, including the overall accuracy, user’s accuracy, producer’s accuracy, and the Kappa coefficient [19,29]. Despite its popularity, the error matrix has been criticized for its inherent uncertainty [19,21,30,31,32,33,34].

A review of remote sensing literature indicates that the accuracy assessment based on the Kappa index is the most widely applied and constitutes the core of the accuracy assessment literature [30,31]. Cohen [35] introduced the Kappa coefficient of agreement to measure the effect and minimize the impact of chance on classifications. This is acknowledged by the remote sensing community as Cohen’s Kappa [36]. The well-known Kappa approach is commonly used to determine the overall accuracy and the proportion of correctly classified pixels. However, Kappa is also often criticized for its inability to indicate the agreement level between the classification and the reference data. The Kappa coefficient measures the level of agreement observed beyond chance, which is computed using a model of chance that is incorrect for the typical accuracy assessment scenario. The fundamental publications that promoted the use of the Kappa coefficient were significant and fostered ideas about rigorous quantitative categorization assessments. However, they promoted an incorrect index [31].

Early research criticized the limitations of Cohen’s Kappa and indicated that Kappa failed to evaluate the inter-rater agreement [17,30,31,37]. Other Kappa versions, such as weighted Kappa and Krippendorff’s Alpha, were proposed to minimize the uncertainty associated with Cohen’s Kappa. Uncertainty of Kappa can be explained as a lake of sureness when one uses it. For example, it is not clear enough how much Kappa can or must be trusted, and it is because of its time-to-time misleading results such as negative Kia values in spite of high accuracy in classification. However, due to the inherent uncertainty and simplicity of computing and interpreting the Cohen’s Kappa, it became the most used method to assess the inter-rater agreement and compute the accuracy in many fields of science, and none of the other proposed indicators succeeded in correcting the accuracy assessment [32]. Kappa has also been incorporated into most of the main image processing software packages (e.g., Erdas, Envi, Idrisi, eCognition, ERmapper). It is also acknowledged that the popularity of Kappa in the remote sensing community results from the software availability and the failure of scientists to appreciate that using percentages as a measure of correctness is a straightforward and easily interpretable alternative [38]. However, the Kappa’s popularity has not led to a reduction in criticisms and documentation of its flaws. The limitations of the Kappa index are obvious when applying it to images classified using the object-based image analysis (OBIA) classification method [18]. OBIA is a soft classification approach that employs fuzzy decision rules as the basis of the image classification. Due to the nature of fuzzy classification, the results of OBIA cannot be easily validated in binary mode and be assigned to ‘true’ and ‘false’ categories. This difficulty results from the segmentation, the scale dependency, rule-based and automated delineation, as well as the broad ontological spectrum related to the object-based classification process [39]. To deal with these issues, researchers have proposed several alternative methods to Kappa, such as the Fuzzy Synthetic Evaluation Dempster–Shafer Theory [17] and spatial accuracy assessment [39]. Our main objective is to introduce a novel and robust accuracy assessment method of quantity and allocation disagreement index (QADI) that resolves the flaws of Kappa and provides an efficient and comprehensive method for accuracy assessment in remote sensing-based classification.

## 2. From Kappa to QADI: The Proposed Approach

### 2.1. Cohen’s Kappa Coefficient

According to Cohen [35], if two independent raters classify each of N objects to one of the n pre-established classes, then the resulting classifications can be displayed in an n×n contingency table with proportions for cell entries, which is also known as the confusion or error matrix [40]. The Kappa coefficient is thus commonly employed to assess the level of agreement between different observers’ ratings or between the same observer’s ratings at various points in time for nominal-level items. The Kappa coefficient (*κ*) can be computed as follows:

(1) $$\kappa =\frac{P_{o}-P_{e}}{1-P_{e}}$$

where $P_{o}=\overset{n}{\underset{i=1}{\sum }}\frac{X_{ii}}{N}$ denotes the observed proportion of agreement and $P_{e}=\overset{n}{\underset{i=1}{\sum }}\frac{X_{+i}}{N}\times \frac{X_{i+}}{N}$ expresses the proportion of chance agreement (expected agreement). Note that all over this article, lowercase n refers to the rank of matrix, while uppercase *N* represents total amount of pixels or observations, and *X_ij_* represents the entry that is placed in row *i* and column *j* of error matrix or raters cross-table (in non-remote-sensing studies). Furthermore, *jk* are temporary integers that change from 1 to *n*. Kappa values range from $\frac{-1}{n-1}$ to +1, where n is the number of classes (rank) and must be greater than 1. A larger *n* value will result in the Kappa index being limited to values between 0 and 1. Cohen interpreted this value of Kappa as the proportion of agreement between the assigners after chance agreement is removed from consideration [40]. A higher Kappa value indicates greater agreement. Kappa is assumed to be positive when agreement exceeds that expected to occur purely by chance. On the other hand, the Kappa value is negative when the observed agreement is less than the expected chance agreement [35]. Table 1 shows guidelines, proposed by Landis and Koch [41], Fleiss et al. [42], and Altman [43], for evaluating the level of agreement in scores based on categorical data.

### 2.2. Criticisms of Kappa

Even though the Kappa index is efficient and popular, it has been criticized and neglected by many researchers. Krippendorff [44] indicated that Cohen’s Kappa should be disqualified as a validation measure for the accuracy assessment of classified thematic maps. This unsuitability for use on thematic maps results from the index’s definition of chance agreement, which is obtained from association measures because it assumes rater independence. Kappa is also criticized for its inherent insufficiency and uncertainty when used for validating image classification results [19,21,30]. Feinstein and Cicchetti [45] reported that one of its primary shortcomings is related to the symmetrically unbalanced situation, called the “first paradox”, which states that “*If p_e_ is large, the chance correction process can convert a relatively high value of p_o_ into a relatively low value of Kappa*” [45]. Furthermore, in the asymmetrical unbalanced situation, for the same p_o_, *κ* will be higher than in the symmetrical unbalanced situation, which is also called the ‘second paradox’. Feinstein and Cicchetti [45] indicated that “*Unbalanced marginal totals produce higher values of κ than more balanced total.”* DiEugenio and Glass [33] also report the same issue in more accessible language: “*κ* is affected by skewed distributions of categories (the prevalence problem) and by the degree to which the coders disagree (the bias problem)”.

### 2.3. The Proposed QADI Index for Accuracy Assessment

**Figure 1:**
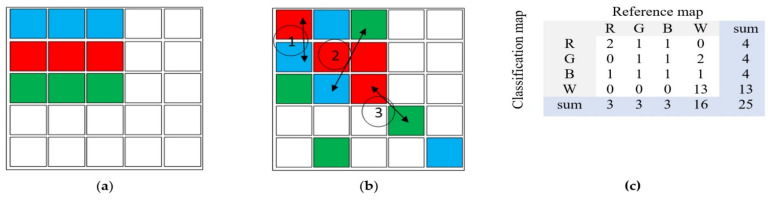
Example to describe the functionality of QADI reference map (**a**), classified map (**b**), and error matrix (**c**).

The quantitative index proposed in this research minimizes the limitations of Cohen’s Kappa by using two types of errors derived from the error matrix (EM): the difference in pixel count between the reference map (RM) and classified map (CM) per class, denoted as Q_i_, and the number of incorrectly labeled pixels, represented by A_i,_. This paper provides a correction that is sometimes needed for the quantity (Q) and allocation (A) indices developed by Pontius and Millones [38]. Then it introduces a new index that summarizes total error level as a unique numeric value and illustrates a graph of classification accuracy. Q_i_ represents the difference in the pixel count between the reference map (RM) and the classified map (CM) for class number (i). This value indicates the ‘quantity disagreement’ between the classification algorithm and the reference map (RM) per class, while ‘Ai’ represents the number of "Required pixel relabelings" that could be interpreted as required movements of pixels to correct their position in sample map below as well as to adapt the classification map (CM) to the reference map (RM) as precisely as possible. Figure 1 shows an example that is useful to describe the functionality of QADI.

As shown in Figure 1, comparing the CM and RM results can be expressed as follows:

(2) $$Q_{1}=\left|4-3\right|=1,\;\;\;\;Q_{2}=\left|4-3\right|=1,\;\;\;\;\;\;Q_{3}=\left|4-3\right|=1,\;\;\;\;\;\;Q_{4}=\left|13-16\right|\;=3\to \;\;\;Q=\frac{1+1+1+3}{2}=3$$

Furthermore, as shown above, movement No. 1 corrects the placement of two pixels, as movement No. 2 does. However, movement No. 3 only corrects the placement of one pixel (green pixel). These three movements make the classified map (CM) much more looking similar to the reference map (RM). Thus, value of A could be calculated as: A=2+2+1=5. 

When the confusion matrix of classification is provided as M, values of Q_i_ and A_i_ for each class number i will be computable as the following formula, which is initially adapted from the formula of Pontius and Millones [38], and accordingly optimized as follows:

(3) $$\forall \;i=1\;:n\;;\;\;Q_{i}=ABS\left(\sum_{k=1}^{n}M_{i,k}-\sum_{k=1}^{n}M_{k,i}\right),\;\;\;\mathrm{and}\;\;\;Q=\frac{\sum_{i=1}^{n}Q_{i}}{2}$$

In some cases, due to reasons such as a skewed distribution, the real value of Q and A might be unexpectedly different from the computation mentioned above. In order to have experimental evidence, two 5 × 5 image models were made from paper. Unlike Pontius and Millones’ [38] article, which uses the size of 3 × 3 and only the two types of black and white pixels, in this study, a 5 × 5 image model as a source image with 25 pixels of colored paper (at least 4 types) has been used. The other 5 × 5 model was a classified image with 25 pixels of colored paper that did not necessarily look similar to the first image because of quantity or label differences (example in Figure 1). By comparing two images in each case, the error matrix of each item was recorded. Then, using the calculations mentioned for Q and A, the obtained numbers from formulas were compared with the reality in the two images. In some cases, for example, the quantity error Q was 5, but in the case of colored-paper pixels, the difference in the number of pixels in the two models was different (more or less). There was a similar condition for labeling (allocation) error as A. According to the initial results of this section, more than a hundred attempts for arranging model pixels and counting place-correction (label-correction) movements of pixels have been considered in the computation trial. Based on these empirical evidences and results, it has been determined that the following adjustments are required:

(4) $$Q^{*}=ABS\left(\sum_{i=1}^{n-1}\sum_{k=1}^{n}M_{i,k}-\sum_{i=1}^{n-1}\sum_{k=1}^{n}M_{k,i}\right).$$

When *Q* differs from Q*^,^ the value of Q* will be used as the quantity disagreement measure, and A must be replaced with $A^{*}=A+ABS\left(Q-Q^{*}\right)$ as the allocation disagreement measure. As shown in Figure 1c, the value of Q^*^ for that matrix equals:

(5) $$\begin{array}{cc} Q^{*}=ABS & \left((\left(2+1+1+0)+(0+1+1+2)+(1+1+1+1\right)\right)\\ & \;\;\;\;\;\;\;\;-(\left(2+0+1+0)+(1+1+1+0)+(1+1+1+0\right)))\\ & \;\;\;\;\;\;\;\;=3. \end{array}$$

Taking a look at both the source and classified images will clarify that this value of 3 is the number of extra pixels that have been added to real numbers of pixels (the difference) in first (n − 1) classes in source image. Rest of pixels do not belong to first (n − 1) class and therefore have same class in both images, and that is why in relation to (4), the value of (n − 1) has been used instead of n. Since this value matches the value of Q that was previously calculated using Equation (5), there is no need to change the value of A and Q to new values. To calculate a value for A_i_, Pontius and Millones [38] proposed the following measures for each i from 1 to n:

(6) $$Omission\;of\;class\;No.\;i=Om_{i}=\sum_{k=1}^{n}\left(M_{k,i}-M_{i,i}\right),\;Commission\;of\;class\;No.\;i=Cm_{i}=\sum_{k=1}^{n}\left(M_{i,k}-M_{i,i}\right),$$

(7) $$A_{i}=2\times min\left\{Om_{i}\;,\;\right.\left.Cm_{i}\right\}.\;\;\mathrm{And},\;\mathrm{finally},\;\;\;\;\;A=\frac{\sum_{i=1}^{n}A_{i}}{2}$$

*M_i,j_* represents the entry of row number *i* and column number *j* of error matrix, and *i,j,k* are temporary integers changing from 1 to n; that is, the rank of matrix.$\;A_{i}$ could be considered as the number of wrongly labeled pixels of class number i or, in other words, as the number of pixels that are not correctly positioned compared to the reference map. Furthermore, each common unit of omission error and commission error for classes implies that if one pixel is misplaced, one other pixel (in a different class) is also misplaced. Therefore, another coefficient must be considered (part *A_i_* in Equation (7)). As a result, all $A_{i}$ factors will be even numbers. Since each pixel movement will correct the placement of one pair, accordingly, the total amount of movements needed to correct all wrongly placed pixels (A) will be equal to the sum of all $A_{i}s$ divided by two (part *A* in Equation (7)). Respecting the idea of Pontius and Millones [38], their index for measuring accuracy $C=1-\left(A+Q\right)$ is only a numerical index such as Kappa, and according to what has been mentioned before, in some skewed distribution cases, it does not correspond to reality, and it needs adjustments. On the other hand, the role of each of the two error types in the total classification error is not clear. The main equation for the QADI index is thus as follows:

(8) $$QADI=\sqrt{{\left(\frac{A}{N}\right)}^{2}+{\left(\frac{Q}{N}\right)}^{2}}$$

The resulting *QADI* value varies between 0 and 1. In order to calibrate the *QADI* index, more than 100 classifications (e.g., land use/cover classification, forest fire mapping, landslide delineation, crop area estimation, etc.) were performed on different satellite images using *OBIA* algorithms, and a confusion matrix was computed for each case. Traditional Kappa and overall accuracy, together with the new *QADI* index, were derived from all error matrices. The graphical representation of *QADI* was produced using Matlab. Table 2 shows the results of the calibration of *QADI* and benchmarks for the strength of the classification accuracy.

**Figure 2:**
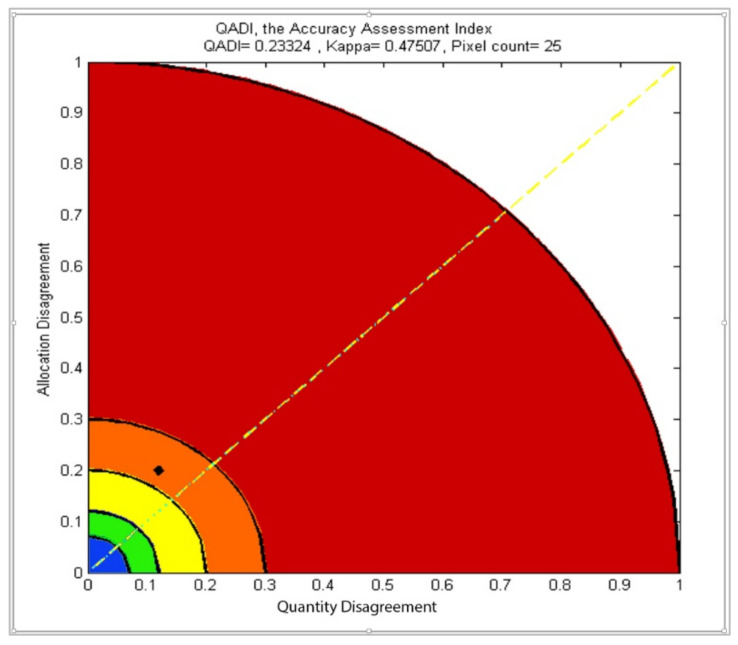
Quantitative analysis of the level of confidence in the computed Kappa.

Using Matlab and Python, which are programing languages, a plugin (Download the tool from Supplementary Materials) was compiled and packed as a toolbox for ArcMap that provides a graph. In addition to the actual QADI value (see Figure 2). The user must not be worried about if the confusion matrix is skewed or not because the algorithm of QADI detects that and will perform the adjustments to Q and A if necessary. In each graph, coordinates (Q/N, A/N) depict a point to illustrate how these factors affect disagreement and which one has a greater impact. Thus, for the image classification experiment shown in Figure 1, the QADI graph illustrates the accuracy as a black dot (Figure 2). As shown in Figure 2, the black dot is located above the diagonal line (yellow line) close to the allocation disagreement axis, which indicates that the primary reason for the disagreement is the allocation error. For this experiment, the numeric QADI value is 0.2332, which indicates an ‘accidental classification’, and that a kind of labeling error (allocation disagreement) has a strong impact on the disagreement and is, in fact, the principal cause of the disagreement. The black dot is above the diagonal line. To be compared, the Kappa value is 0.4751, which implies an ‘intermediate to good’ classification according to the benchmark proposed by Landis and Koch [41]. Further, it implies a ‘moderate classification’ according to the benchmark proposed by Fleiss et al. [42] and Altman [43]. The proposed approach allows computing the error matrix and interpreting the efficiency of the computed Kappa based on the QADI graph and value. The QADI graph, shown in Figure 2, enables a quantification of the confidence level in the computed Kappa. The QADI value also theoretically varies in the range of 0–1, as indicated in Table 2.

### 2.4. The Validation Experiments

In order to examine the QADI index, its functionality and performance were evaluated and compared against the traditional Kappa index. Therefore, two experiment classifications using various algorithms were established. The first one has a balanced distribution, and the other has a skewed distribution of 500 pixels each (see Table 3). The classifications were categorized into four classes: water body, soil, vegetation, and urban area. In order to compare the results of QADI and Kappa, confusion matrices for both classifications were derived and provided in Table 3. According to these confusion matrices, both classifications determined the same proportion (80%) of correctly classified pixels. The rater A (first rater) is the reference map, and the rater B (second rater) is the classification algorithm.

For the balanced distribution, as shown in the confusion matrix on the Table 3a, the sums of the lines are 124, 125, 125, 126, and the sums of the columns are 124, 124, 126, 126, respectively. According to this confusion matrix, the sums of the lines and columns do not show significant differences, and they are within a similar range, which clearly indicates that this confusion matrix has a balanced distribution. However, for the confusion matrix with a skewed distribution (Table 3b), the sums of the lines are 445, 44, 8, 3, and the sums of the columns are 445, 44, 8, 3, respectively. As this confusion matrix shows, the obtained values are not in the same range, which clearly indicates a skewed distribution in the confusion matrix. In this experiment, 500 pixels were considered for classification, and according to both confusion matrices, 400 pixels (80%) were classified correctly. Since the classification was performed based on the same decision rules, it is anticipated that Kappa will deliver the same accuracy for both confusion matrices. We may highlight that Kappa is an index that is computed from confusion matrices. Despite having the same accuracy for both confusion matrices, Kappa represented an accuracy of 73% for the confusion matrix with a balanced distribution and a negative value for the confusion matrix with a skewed distribution. This shows that Kappa is very sensitive to the distribution in the confusion matrix, and it may even deliver misleading results for a confusion matrix with a skewed distribution. The sensitivity of Kappa to the distribution in the confusion matrix is the main issue with Kappa and the primary issue that the proposed QADI method aims to overcome. In order to confirm this superiority of the QADI method, a step-by-step computation of the QADI index is derived separately for both confusion matrices (balanced and skewed distribution) and represented in the following sections:

### 2.5. Confusion Matrix with a Balanced Distribution

As stated in relation (1), the factors required to determine Kappa and the κ value are calculated as follows:

(9) $$P_{o}=\overset{n}{\underset{i=1}{\sum }}\frac{X_{ii}}{N}=\frac{100+100+100+100}{500}=0.8\;P_{e}=\overset{n}{\underset{i=1}{\sum }}\frac{X_{+i}}{N}\times \frac{X_{i+}}{N}=\frac{124}{500}\times \frac{124}{500}+\frac{124}{500}\times \frac{125}{500}+\frac{126}{500}\times \frac{125}{500}+\frac{126}{500}\times \frac{126}{500}=0.25\;\to \kappa =\frac{P_{o}-P_{e}}{1-P_{e}}=\frac{0.8-0.25}{1-0.25}=0.73$$

Quantity disagreement of classes:

(10) $$Q_{1}=\left|124-124\right|=0,\;Q_{2}=\left|124-125\right|=1,\;Q_{3}=\left|126-125\right|=1,\;Q_{4}=\left|126-126\right|=0.\;\to Q=\frac{0+1+1+0}{2}=1.$$

Omission and commission of classes:

$$Om_{1}=124-100=24,\;Om_{2}=124-100=24,\;Om_{3}=126-100=26,\;Om_{4}=126-100=26\;Cm_{1}=124-100=24,\;Cm_{2}=125-100=25,\;Cm_{3}=125-100=25,\;Cm_{4}=126-100=26$$

Allocation disagreement of classes according to Equation (7):

(11) $$A_{1}=2\times min\left\{Om_{1}\;,\;\right.\left.Cm_{1}\right\}\to A_{1}=2\times min\left\{24,\right.\left.24\right\}=48.$$

In the same way, $A_{2}=48,\;A_{3}=50,\;A_{4}=52$ and so the total allocation disagreement is equal to: $A=\frac{48+48+50+52}{2}=99.$

To ensure that *A* and *Q* are calculated correctly, $Q^{*}$ must be calculated:

(12) $$Q^{*}=ABS(\left(\left(100+8+8+8\right)+\left(8+100+8+9\right)+\left(8+8+100+9\right)\right)\;\begin{array}{c} -\;(\left(100+8+8+8\right)+\left(8+100+8+8\right)\\ +\left(8+8+100+10\right)))=0 \end{array}$$

Since $Q^{*}$ differs from $Q^{\;},$ the value of the quantity disagreement should be set to 0, and the value of the allocation disagreement should be set to $99+ABS\left(1-0\right)=100$.

Finally, the QADI will be:

(13) $$QADI=\sqrt{{\left(\frac{100}{500}\right)}^{2}+{\left(\frac{0}{500}\right)}^{2}}=0.2$$

According to the interpretation presented in Table 1, this value implies “Poor Accuracy” of the developed classification.

### 2.6. Confusion Matrix with a Skewed Distribution


(14) $$P_{o}=\overset{n}{\underset{i=1}{\sum }}\frac{X_{ii}}{N}=\frac{400+0+0+0}{500}=0.8$$

(15) $$P_{e}=\overset{n}{\underset{i=1}{\sum }}\frac{X_{+i}}{N}\times \frac{X_{i+}}{N}=\frac{445}{500}\times \frac{445}{500}+\frac{44}{500}\times \frac{44}{500}+\frac{8}{500}\times \frac{8}{500}+\frac{3}{500}\times \frac{3}{500}=0.800136$$

(16) $$\kappa =\frac{P_{o}-P_{e}}{1-P_{e}}=\frac{0.8-0.800136}{1-0.800136}=-0.00068$$

Quantity disagreement of classes:

(17) $$Q_{1}=\left|445-445\right|=0,\;Q_{2}=\left|44-44\right|=0,\;Q_{3}=\left|8-8\right|=0\;and\;Q_{4}=\left|3-3\right|=0.\;\to Q=\frac{0+0+0+0}{2}=0$$

Omission and commission of classes:

$$Om_{1}=445-400=45,\;Om_{2}=44-0=44,\;Om_{3}=8-0=8\;and\;Om_{4}=3-0=3$$

$$Cm_{1}=445-400=45,\;Cm_{2}=44-0=44,\;Cm_{3}=8-0=8\;and\;\;Cm_{4}=3-0=3$$

Allocation disagreement of classes according to Equation (7):

(18) $$A_{1}=2\times min\left\{Om_{1}\;,\;\right.\left.Cm_{1}\right\}\to A_{1}=2\times min\left\{45,\right.\left.45\right\}=90$$

In the same way, $A_{2}=88,\;A_{3}=16,\;A_{4}=6$ and so the total amount of allocation disagreement is equal to: $A=\frac{90+88+16+6}{2}=100.$

To ensure that *A* and *Q* are calculated correctly, $Q^{*}$ must be calculated:

(19) $$Q^{*}=ABS(\left(\left(400+40+4+1\right)+\left(40+0+3+1\right)+\left(4+3+0+1\right)\right)\;\begin{array}{c} -(\left(400+40+4+1\right)+\left(40+0+3+1\right)\\ +\left(4+3+0+1\right)))=0 \end{array}$$

Since $Q^{*}$ does not differ from Q, the values of the quantity disagreement and the allocation disagreement are correct. Finally, QADI will be equal to:

(20) $$QADI=\sqrt{{\left(\frac{100}{500}\right)}^{2}+{\left(\frac{0}{500}\right)}^{2}}=0.2$$

**Figure 3:**
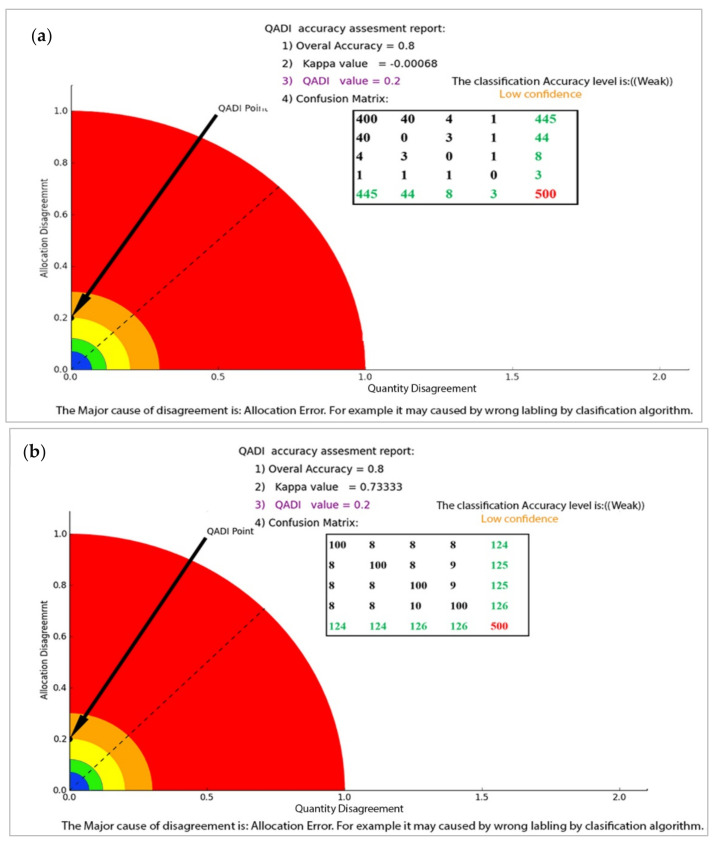
Accuracy assessment illustrated by the graphical representation of QADI. (**a**) QADI graph for Skewed matrix and (**b**) QADI graph for normal matrix2.7. Implementation and use cases.

According to the interpretation presented in Table 1, this value implies “Poor Accuracy” of the classification, as does the QADI value for the balanced distribution. Figure 3 shows two samples of normal and skewed matrices, as represented in Table 3. Figure 3a shows that in the case of the skewed matrix and its respective QADI graph, Kappa is subject to the first paradox issue by representing a negative value of −0.00068, which is strange provided the 80% overall accuracy. Contrarily, the proposed QADI computed the classification accuracy to be 0.2, which indicates a ‘low confidence’. In addition, the QADI also interpreted the classification error as incorrect labeling resulting from the classification algorithm. Figure 3b represents the normal error matrix and the QADI graph. Here, Kappa indicates a moderate accuracy of 0.73333, while the QADI considers it as low confidence.

**Figure 4:**
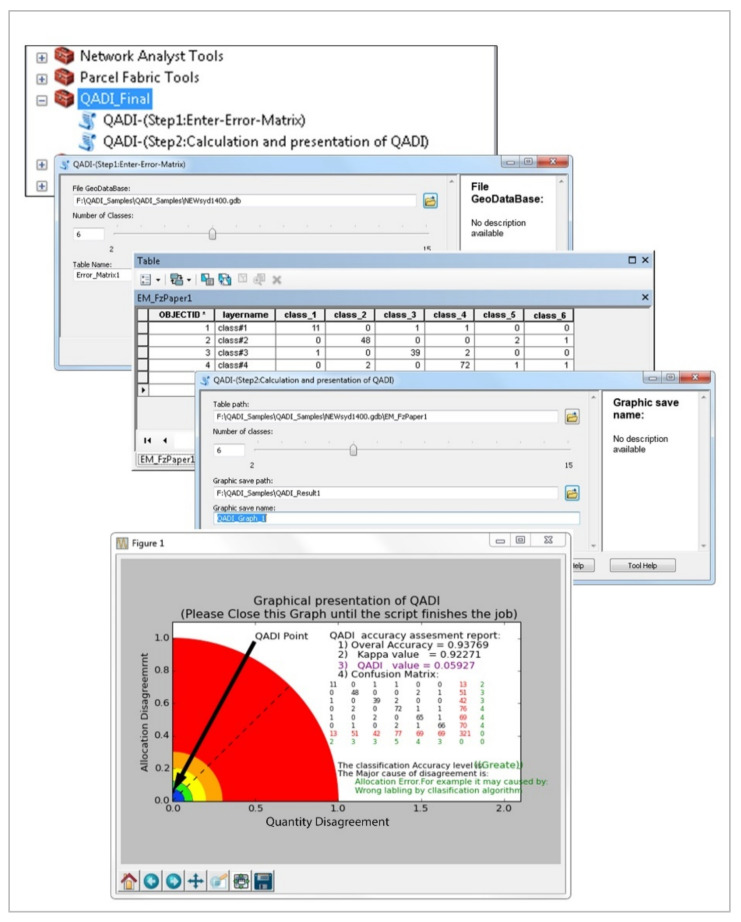
Toolbox developed for the implementation of QADI.

**Figure 5:**
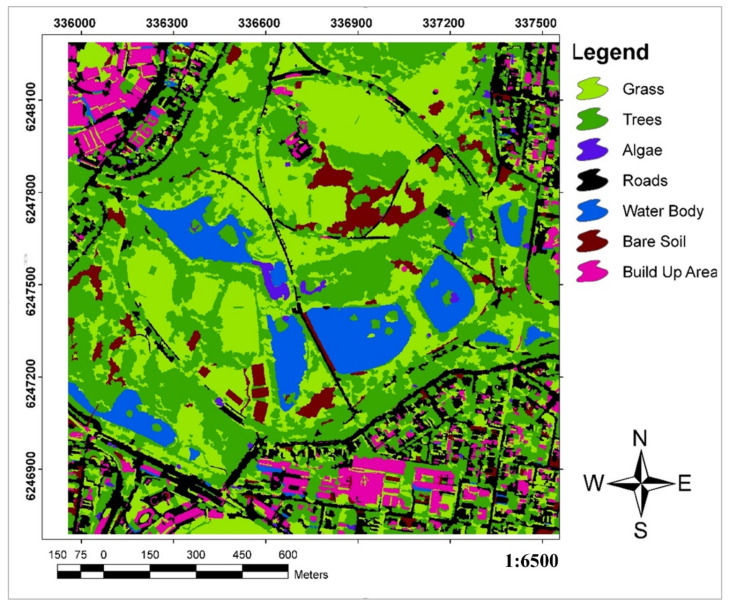
Results of LULC classification for Sydney.

To apply the proposed QADI approach, we developed a toolbox in Python (Figure 4) that allows the calculation of QADI in a GIS environment. Several experiments were conducted to examine the efficiency of the proposed QADI approach for accuracy assessment. The first case is an object-based image analysis (OBIA) land use land cover (LULC) classification for a subset of Sydney, Australia. The LULC map was produced based on OBIA using different techniques based on each image’s context and the LULC classes (Table 4 summarizes the object-based rule set for the LULC classifications). For this goal, we collocated ground control point data for each class; accordingly, 70% of all data were employed as training data, and 30% employed for validation task. Figure 5 shows the developed LULC map and its respective error matrix based on the ground control points provided in Table 5. In addition to calculating the overall accuracy and Kappa value, the error matrix was stored to be used in the QADI calculator plugin. We also used five error matrices from earlier work and research literature as input for the QADI to examine its efficiency. Therefore, we considered error matrices for the accuracy assessment of the LULC classification using different data-driven approaches, namely OBIA (Table 6: [46]), deep learning (Table 7: [47]), and the three machine learning algorithms random forest, support vector machine, and artificial neural network (Table 8, Table 9 and Table 10: [48]).

## 3. Results

This section presents the results of using the QADI method for the accuracy assessment of several LULC classifications, as discussed in the implementation section. Results of this investigation show that in addition to previous defections (e.g., skewness sensitivity and the paradoxes), the numeric Kappa index cannot determine the causes of disagreement between classification maps and the reference maps. A summary of the results is presented in Table 11 to provide a better comparison between the performance of the Kappa and QADI indexes. This shows that Kappa provides a result for a skewed distribution dataset that fails the common-sense test and confirms the work of various researchers who have already criticized Kappa [45,49,50,51].

**Figure 6:**
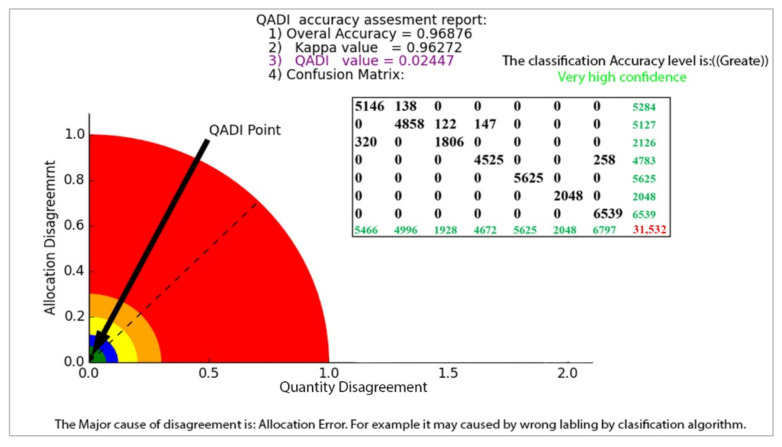
The QADI graph for the error matrix of the Sydney LULC classification.

As discussed in the implementation section, we examined the efficiency of the proposed QADI method in several case studies. Figure 6 presents the QADI graph developed based on the error matrix in Table 5 for a Sydney LULC classification case study. The obtained Kappa for this error matrix was 0.96, which, as Altman [43] pointed out, can be considered a very high and accurate classification. As indicated, the QADI value for this classification is computed to be 0.024, which indicates a “very high confidence” in the classification. Based on the QADI graph, the QADI point is located within the blue area and thus expresses that the classification error is caused by incorrect labeling, which might be caused by the classification algorithm.

**Figure 7:**
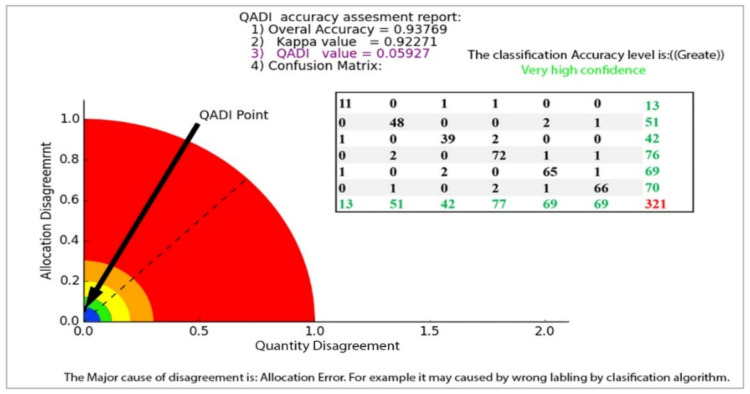
The QADI graph for the error matrix of the OBIA-based LULC classification (Table 6) by Naboureh et al. [46].

**Figure 8:**
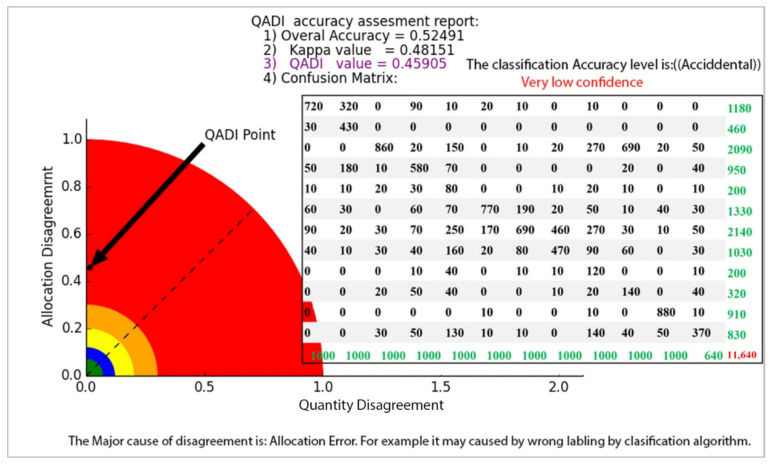
The QADI graph for the error matrix for Table 7 developed by Rousset et al. [47].

**Figure 9:**
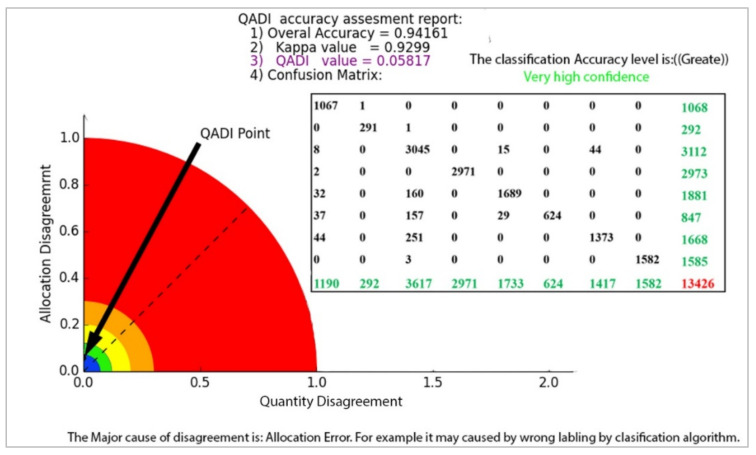
The QADI graph for the error matrix of LULC classification based on RF, (Table 8) developed by Leeuwen et al. [48].

**Figure 10:**
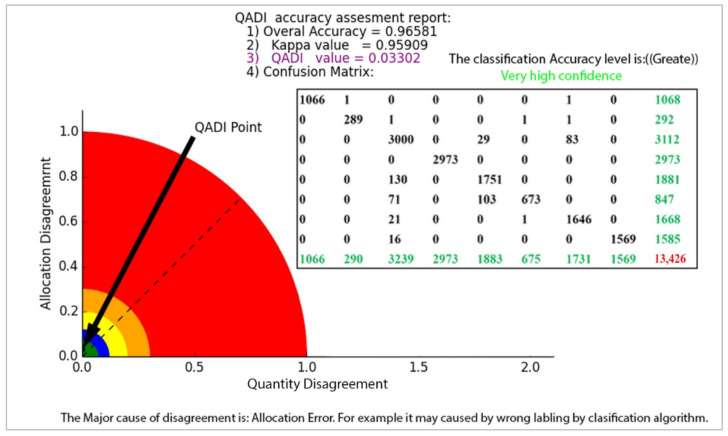
The QADI graph for the error matrix of LULC classification based on SVM (Table 9) developed by Leeuwen et al. [48].

**Figure 11:**
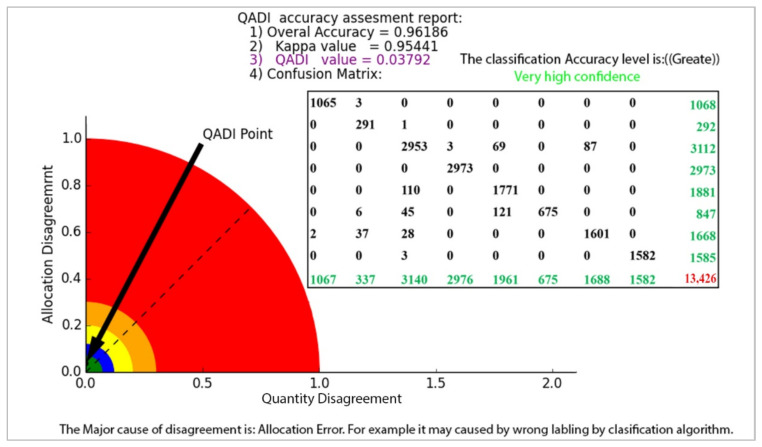
The QADI graph forthe error matrix of LULC classification based on ANN (Table 10) developed by Leeuwen et al. [48].

The QADI graph for the OBIA-based LULC classification matrix provided in Table 6 [46] is presented in Figure 7. The Kappa for this error matrix was 0.92271, and, based on the graph, the QADI point with a value of 0.05927 is in the green area, which indicates a “very high confidence” for the classification. Both indexes indicated the classification to be efficient based on the Altman [43] scale. In addition, the QADI was also able to deduce the cause of the classification error to be incorrect labeling. Figure 8 presents the QADI graph for the error matrix in Table 7 [47]. The Kappa value for this matrix is 0.48, and the computed QADI value is 0.45905 and lies within the red area, which indicates a very low confidence. Based on the QADI result indicating the labeling error as the cause of the low accuracy, the user could improve the accuracy by applying efficient classification algorithms. As previously indicated, we also used the accuracy assessment results for LULC classification based on the three machine learning techniques of RF, SVM and ANN previously published by Leeuwen et al. [48]. Figure 9, Figure 10 and Figure 11 depict the QADI values for the error matrices for the accuracy assessments of LULC classifications computed to be 0.05817, 0.03302, and 0.03792, respectively, all of which lie within the green area and thus indicate a very high confidence.

## 4. Discussion

### 4.1. Kappa Index and Issues

In terms of the accuracy assessment of thematic maps derived from remote sensing data, when two or more raters that are equally skilled categorize the same observations or objects into specific and separate pre-defined classes, there is a keen interest in knowing the level of agreement between a classified map and a reference map or between different classifications. As state of art, the main objective of this research was to develop and propose an alternative method to the Kappa index for accuracy assessment. Results of implementation in several experiments indicate that the QADI is an effective index to investigate the accuracy of thematic and classified maps. According to Ye et al. [52], a literature review shows that Kappa has been used in at least 40% of the remote sensing-based published papers between 2003 and 2017. Despite the popularity of Kappa, the results acknowledged that Kappa, due to its statistical orientation, is prone to uncertainty in decisions and may not be able to efficiently evaluate the accuracy of thematic maps. Pontius and Millones [38] and Foody [31] demonstrate why Kappa is a poor statistic for accuracy assessment regardless of whether the confusion matrix is derived from OBIA or some other categorization. Kappa compares the measured accuracy to a sometimes deceptive and often irrelevant random accuracy baseline, especially when dealing with a skewed matrix, as represented in Table 3 and Figure 3. Because random categorization is usually not an alternative approach for creating the map, a comparison to random accuracy is usually meaningless. Even if random categorization were important, Kappa calculates random accuracy incorrectly. Technically, a totally random classification would yield a correct proportion equal to one divided by the number of categories [19], which is not the Kappa baseline.

Furthermore, Kappa muddles quantity and allocation disagreements in a way that makes interpretation difficult [14,38,52]. Values that span the full range of widely used interpretation scales, indicating a level of agreement that equates to that estimated to arise from chance alone all the way through to nearly perfect agreement, can be obtained from classifications that meet demanding accuracy targets [31]. Thus, the error measures obtained from the confusion matrix will not represent the map attributes if the class proportions in the confusion matrix fail to represent the actual landscape proportions. If testing samples are created using an entirely random sample, the proportion of samples in each class will provide an unbiased estimate of the map’s characteristic attributes. However, the analytic approach will be different if alternative sampling methods, such as stratified random sampling, are used [18].

### 4.2. Significance of QADI

Recent progress in earth observation and remote sensing technologies produced satellite images with improved spatial, spectral, and temporal resolutions, which accordingly increased the demands of efferent data-driven approaches [53,54,55,56]. In this context, a number of machine learning and particularly deep learning methods were developed and proposed over the past decade [13]. Despite developing a variety of classification methods (e.g., machine learning, deep learning, etc.), the accuracy assessment methods are still under-developed, and it remains an area of active research, and disagreements on key accuracy assessment issues remain. Most derived measures, including the overall accuracy, are generated from the confusion matrix. The common purpose of these accuracy measures is to describe the correctness of maps that are intended to reflect real landscapes. In addition, the traditional accuracy assessment methods such as Kappa were criticized by the remote sensing community, and their efficiency for the navel data-driven approaches is still a question of interest. Thus, our proposed QADI index can be employed for data-driven approaches such as OBIA, pixel-based, machine learning, etc. In this context, we examined its efficiency by analyzing the accuracy of different data-driven approaches, including OBIA (Table 5 and Table 6), deep learning (Table 7), random forest (Table 8), support vector machine (Table 9), and artificial neural network (Table 10). The obtained results indicated the capability of QADI as an efficient accuracy assessment for a variety of data-driven approaches. In addition, the advantage of QADI is that this method resolves the issues of the traditional Kappa index by means of a numeric and graphic representation of accuracy.

The numeric values of the QADI index range from 0 to 1, whereby lower QADI values represent a higher accuracy. QADI measures the number of disagreements between the reference map and the classified map. Technically, disagreement depends on two factors, namely quantity disagreement (*Q*) and allocation disagreement (*A*). Aside from representing a numeric index, QADI can also be used to illustrate the classification accuracy graphically. The graphical representation of QADI depicts which factors have a stronger contribution to the disagreement and uncertainty of the results. The proposed QADI also provides the statement for the obtained QADI value. The graphical QADI representation can be used to further investigate and thus improve the methods and algorithms that are employed for image classification. The results clearly indicate that the QADI index is not sensitive to the variety of distribution in the confusion matrix and does not allow the variety in the confusion matrix to impact the results. Especially in OBIA, QADI leads to an improvement of the rule-sets that are applied for image classification. This specific suitability of QADI for OBIA is based on the advantage that QADI can be applied to determine “how and to what extent quantity or labeling errors may occur when applying each rule-set or classification algorithm to an image”. Furthermore, QADI solves some problematic paradoxes faced by the Kappa index. Especially skewed distributions that cause irregular Kappa values are treated in a more precise manner by QADI.

## 5. Conclusions

Remote sensing has become a critical technology for environmental monitoring and application. Based on recent progress in earth observation technologies and the availability of a variety of improved satellite images, data-driven methods (e.g., Machine learning, deep learning, etc.) have been proposed in the remote sensing domain. The Kappa index is popular due to its availability in image processing programs and its easy implementation. Since the accuracy assessment is a critical step for validating the thematic maps derived from remote sensing, and the issues associated with the traditional Kappa method have already been demonstrated, introducing the new and effective QADI method is a significant contribution to the domain of remote sensing sciences. The results of this research demonstrated that the QADI index could be employed as an efficient alternative to Kappa. Because of the functionality and efficiency of this method, we intend to publish the developed method and its implementation toolbox as an open-source toolbox to support future studies. We also share the toolbox and codes with image processing software companies (e.g., eCognation, Erdas, Envi) for future extension of the accuracy assessment methods in most applied software. We consider this to be progressive research furthering the field of remote sensing. Considering the increase in remote sensing and its applications in different fields and the availability of different data-driven approaches, the proposed QADI will benefit the remote sensing community as a novel state-of-the-art accuracy assessment method. It is very important to mention this point that QADI has been developed to work on error matrixes and, therefore, it is not dependent on methodology for the classification of images. In fact, any method or algorithm such as OBIA, pixel-based, machine learning, or deep learning methodology that has the capability to classify satellite images and produce a confusion matrix (error matrix) may be analyzed by QADI much more efficiently. As state of the art, we conclude that the results of this research are of great importance from a methodological perspective for any validation and accuracy assessment task and will significantly contribute to progressing the state of research in remote sensing itself and in its role as a cross-cutting interdisciplinary field. Based on the results of this research and our early studies for developing a navel methodology for accuracy assessment [17], our future research will focus on integrating the QADI with spatial uncertainty analysis methods such as Dempster–Shafer Theory to improve the efficiency of QADI as outstanding accuracy assessment for remote sensing. We conclude that the proposed QADI can be employed as a base of accuracy assessment method in remote sensing.

## Figures and Tables

**Table 1:** Thresholds for the strength of agreement that have been constructed for Kappa in literature.

Landis and Koch Benchmark Scale for the Kappa Index		Fleiss’s Benchmark Scale for the Kappa Index		Altman’s Benchmark Scale for the Kappa Index	
<0.40 0.40 to 0.75 More than 0.75	Poor Intermediate to good Excellent	<0.0	Poor	<0.20	Poor
		0.21 to 0.40	Fair	0.21 to 0.40	Fair
		0.41 to 0.60	Moderate	0.41 to 0.60	Moderate
		0.61 to 0.80	Substantial	0.61 to 0.80	Good
		0.81 to 1.00	Almost perfect	0.81 to 1.00	Very good

**Table 2:** Calibration for QADI and benchmarks for the strength of the classification accuracy.

QADI Scale	Color Scheme	Classification Accuracy
0.00≤QADI<0.07	Blue	Very high confidence/(Very low disagreement)
0.07≤QADI<0.12	Green	High confidence/(Low disagreement)
0.12≤QADI<0.20	Yellow	Moderate confidence/(Moderate disagreement)
0.20≤QADI<0.30	Orange	Low confidence/(High level of disagreement)
0.30≤QADI≤1	Red	Very low confidence/(lack of accuracy)

**Table 3:** Tables illustrating a confusion matrix with a balanced (a) and a skewed (b) distribution.

(a) Balanced distribution					
**Rater B** **(Practice)**	**Rater A (Reference Map)**				**Sum**
	**W**	**S**	**V**	**U**	
Water body	100	8	8	8	124
Soil	8	100	8	9	125
Vegetation	8	8	100	9	125
Urban area	8	8	10	100	126
sum	124	124	126	126	500
(b) Skewed distribution					
**Rater B** **(Algorithm)**	**Rater A (Reference Map)**				**Sum**
	**W**	**S**	**V**	**U**	
Water	400	40	4	1	445
Soil	40	0	3	1	44
Vegetation	4	3	0	1	8
Urban	1	1	1	0	3
sum	445	44	8	3	500

**Table 4:** Details of the OBIA-based LULC classification for the Sydney use case.

Satellite images	Resolution of 1.6 m
Segmentation parameters	Scale of 30, shape index of 0.8 and compactness of 0.5
LULC classes	Grass, Trees, Algae, Roads, Water body, Built up area, Bare soil
Features and algorithms	Shape indexes, GLCM textural parameters, normalized difference vegetation index (0.24> and <0.3), ratio of green (<0.3), length/width (0.9>), rectangular fit indexes (1.3–1.6 and 0.3–0.05), shape indexes, GLCM textural parameters, normalized difference vegetation index (0.3> and <0.8), ratio of green (0.4>), brightness (135>), length/width (0.9>), rectangular fit (1.2–1.5), mean (1.6>)
Classification algorithm	Sample-based supervised classification based on nearest neighbor
Accuracy assessment	Control points for the error matrix and to calculate the Kappa and QADI

**Table 5:** Error matrix for the accuracy assessment of the Sydney LULC classification.

User/Reference	Grass	Trees	Algae	Roads	Water Body	Bare Soil	Built up Area	Sum
Grass	5146	138	0	0	0	0	0	5284
Trees	0	4858	122	147	0	0	0	5127
Algae	320	0	1806	0	0	0	0	2126
Roads	0	0	0	4525	0	0	258	4783
Water body	0	0	0	0	5625	0	0	5625
Bare Soil	0	0	0	0	0	2048	0	2048
Built up area	0	0	0	0	0	0	6539	6539
Column total	5466	4996	1928	4672	5625	2048	6797	31,532
Overall accuracy	0.97							
Kappa	0.93							

**Table 6:** Error matrix for accuracy assessment of LULC classification using OBIA (Naboureh et al. [46]).

User/Reference	W	RA	SL	FA	BL	OIA	Sum
Water(W)	11	0	1	1	0	0	13
Residential area (RA)	0	48	0	0	2	1	51
Salty lands (SL)	1	0	39	2	0	0	42
Farm agriculture (FA)	0	2	0	72	1	1	76
Bare lands (BL)	1	0	2	0	65	1	69
Orchard and irrigated agriculture (OIA)	0	1	0	2	1	66	70
Column total	13	51	42	77	69	69	320
Accuracy	0.84	0.94	0.92	0.93	0.94	0.95	
Overall Accuracy = 0.94							
Kappa = 0.92							

**Table 7:** Error matrix for accuracy assessment of LULC classification based on deep learning (Rousset et al. [47]).

	a	b	c	d	e	f	g	h	i	j	k	l
Urban areas (a)	720	320	0	90	10	20	10	0	10	0	0	0
Industrial Areas (b)	30	430	0	0	0	0	0	0	0	0	0	0
Worksites and mines (c)	0	0	860	20	150	0	10	20	270	690	20	50
Road Networks (d)	50	180	10	580	70	0	0	0	0	20	0	40
Trails (e)	10	10	20	30	80	0	0	10	20	10	0	10
Forests (f)	60	30	0	60	70	770	190	20	50	10	40	30
Medium-density Vegetation (g)	90	20	30	70	250	170	690	460	270	30	10	50
Low-density vegetation (h)	40	10	30	40	160	20	80	470	90	60	0	30
Bare rocks (i)	0	0	0	10	40	0	10	10	120	0	0	10
Bare soil (j)	0	0	20	50	40	0	0	10	20	140	0	40
Water surfaces (k)	0	0	0	0	0	10	0	0	10	0	880	10
Engravements (l)	0	0	30	50	130	10	10	0	140	40	50	370
Column total												
Overall accuracy	0.52											
Kappa	0.48											

**Table 8:** Error matrix for accuracy assessment of LULC classification using random forest (Leeuwen et al. [48]).

LULC	Deep Water	Shallow Water	Urban	Bare Soil	Agricultural Land	Grassland	Forest	Cloud	Sum
Deep water	1067	1	0	0	0	0	0	0	1068
Shallow water	0	291	1	0	0	0	0	0	292
Urban	8	0	3045	0	15	0	44	0	3112
Bare soil	2	0	0	2971	0	0	0	0	2973
Agricultural land	32	0	160	0	1689	0	0	0	1881
Grassland	37	0	157	0	29	624	0	0	847
Forest	44	0	251	0	0	0	1373	0	1668
Cloud	0	0	3	0	0	0	0	1582	1585
Sum	1190	292	3617	2971	1733	624	1417	1582	13,426
Overall accuracy	0.94								
Kappa	0.93								

**Table 9:** Error matrix for accuracy assessment of LULC classification using support vector machine (Leeuwen et al. [48]).

LULC	Deep Water	Shallow Water	Urban	Bare Soil	Agricultural Land	Grassland	Forest	Cloud	Sum
	1066	1	0	0	0	0	1	0	1068
Shallow water	0	289	1	0	0	1	1	0	292
Urban	0	0	3000	0	29	0	83	0	3112
Bare soil	0	0	0	2973	0	0	0	0	2973
Agricultural land	0	0	130	0	1751	0	0	0	1881
Grassland	0	0	71	0	103	673	0	0	847
Forest	0	0	21	0	0	1	1646	0	1668
Cloud	0	0	16	0	0	0	0	1569	1585
Sum	1066	290	3239	2973	1883	675	1731	1569	13,426
Overall accuracy	0.97								
Kappa	0.96								

**Table 10:** Error matrix for accuracy assessment of LULC classification using artificial neural network (Leeuwen et al. [48]).

LULC	Deep Water	Shallow Water	Urban	Bare Soil	Agricultural Land	Grassland	Forest	Cloud	Sum
Deep water	1065	3	0	0	0	0	0	0	1068
Shallow water	0	291	1	0	0	0	0	0	292
Urban	0	0	2953	3	69	0	87	0	3112
Bare soil	0	0	0	2973	0	0	0	0	2973
Agricultural land	0	0	110	0	1771	0	0	0	1881
Grassland	0	6	45	0	121	675	0	0	847
Forest	2	37	28	0	0	0	1601	0	1668
Cloud	0	0	3	0	0	0	0	1582	1585
Sum	1067	337	3140	2976	1961	675	1688	1582	13,426
Overall accuracy	0.96								
Kappa	0.95								

**Table 11:** Comparison of QADI and Kappa values for a balanced distribution and a skewed distribution.

Confusion Matrix with a Balanced Distribution		Confusion Matrix with a Skewed Distribution	
Kappa	0.73	Kappa	−0.00068
QADI	0.2	QADI	0.2

## Data Availability

The source code (QADI toolbox for ArcMap) is available for downloading at the link: https://drive.google.com/file/d/1lMDVknlfFFWDC5k1F0GVQpcb7vJfRwEK/view.

## Supplementary Materials

The following supporting information can be downloaded at: https://www.mdpi.com/article/10.3390/s22124506/s1.
