# Supplementary material for: QADI as a New Method and Alternative to Kappa for Accuracy Assessment of Remote Sensing-Based Image Classification
Source: Sensors (Basel). 2022 Jun 14;22(12):4506. doi: 10.3390/s22124506 (PMC9230807; doi:10.3390/s22124506)
Supplement: Supplementary file 1 [file sensors-22-04506-s001.zip › QADI Toolbox/QADI_Toolobox_Help.pdf]

How to use QADI toolbox?

First of all, thank you for using QADI. This toolbox has been scripted in Python and turned into an ArcMap toolbox. Users that aim to assess classifications accuracy (confidence) may use it. Following steps are necessary to use QADI toolbox:

I. Adding toolbox to ArcMap

Run ArcMap and right-click in ArcToolbox, select "Add Toolbox…" and then select the path you extracted QADI_Tool.rar. There will be a file named "QADI_Final_Ver1.0.tbx". Select it and click "Open". You will see QADI_Final in ArcToolbox.

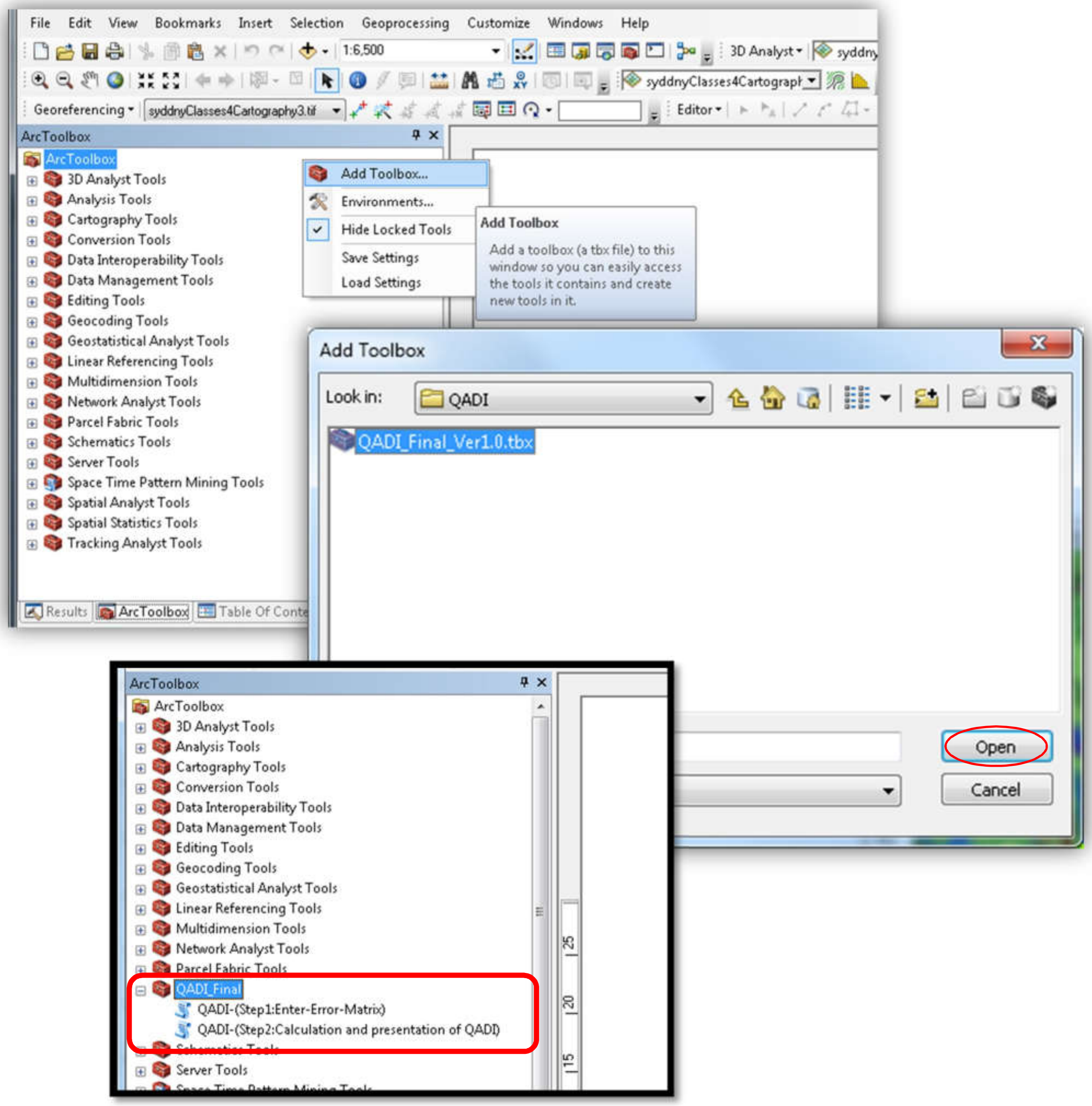

II. Running QADI toolbox in ArcMap (Step 1)

As you can see, QADI toolbox included two steps. First step will acquire the Error matrix rank (Number of classes) from user and will make an empty matrix in user defined path. There must be an existing file geo database to choose or made a new one by clicking 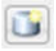 icon.

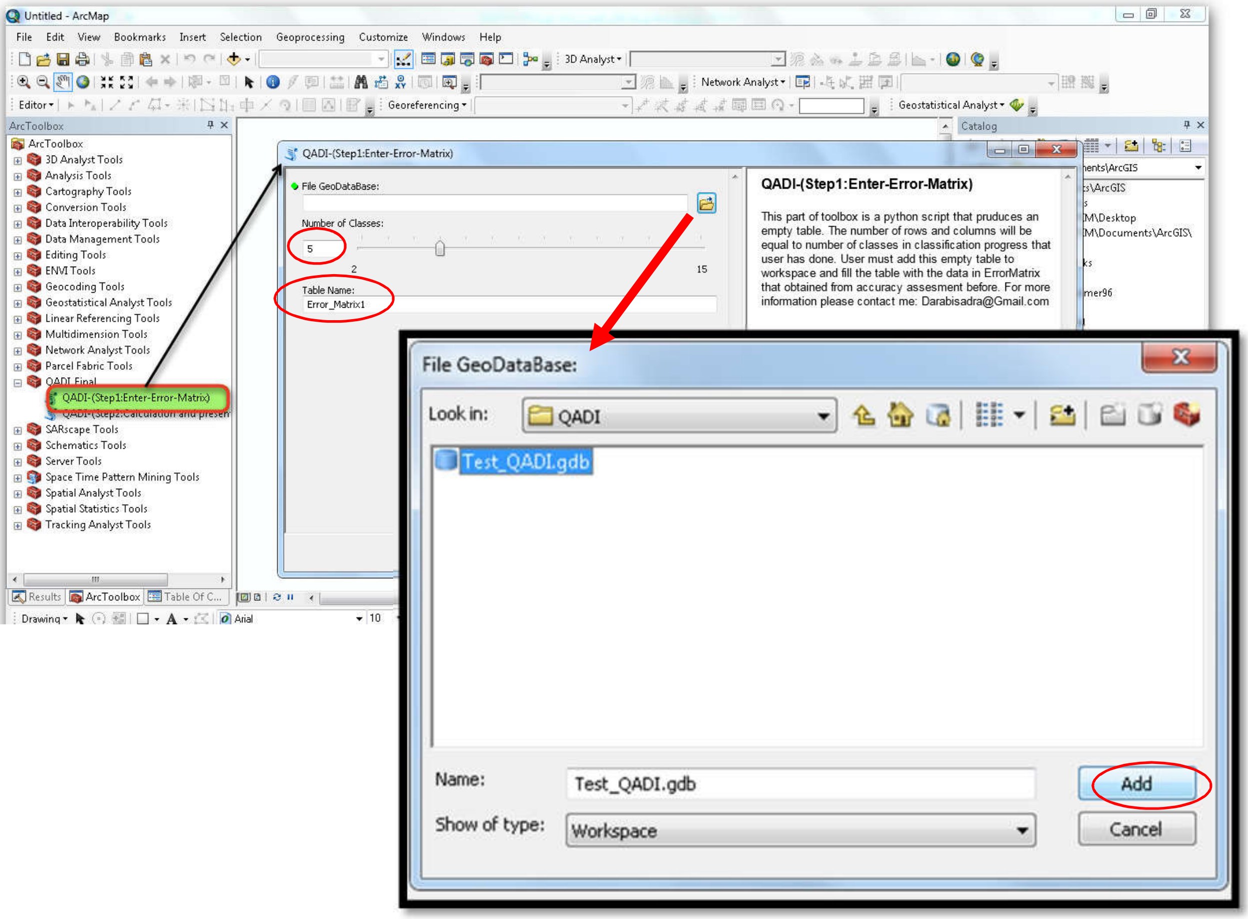

Few seconds later a message will appear and announces that script has been completed successfully and it means that the table is ready.

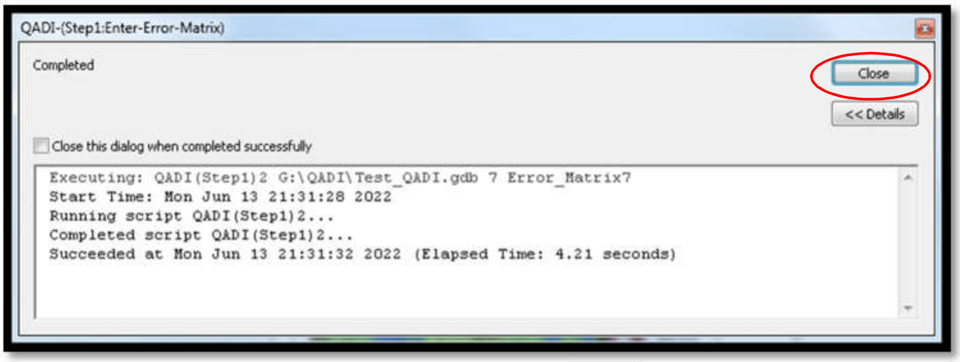

User must add that empty table to ArcMap. Click on 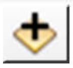 icon and select the path you entered to make table in first step.

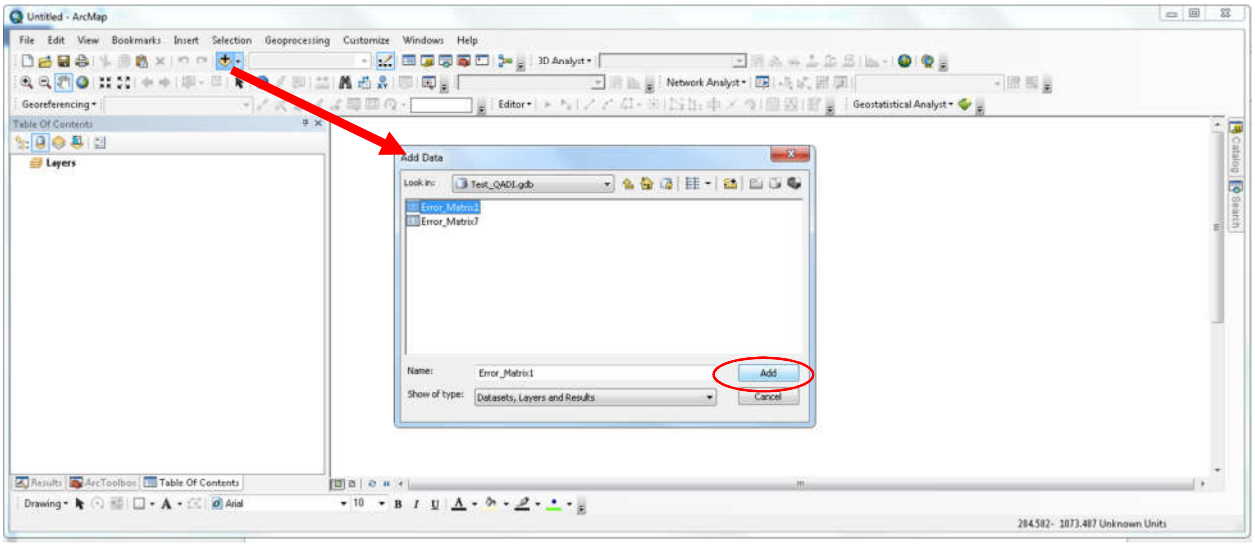

Take a look at "Table of Contents" and you will see the table, right click on it and select open. All entries of matrix (The table) are set as "Null". They must be replaced with entries of error-matrix that achieved from accuracy assessment process and has been saved.

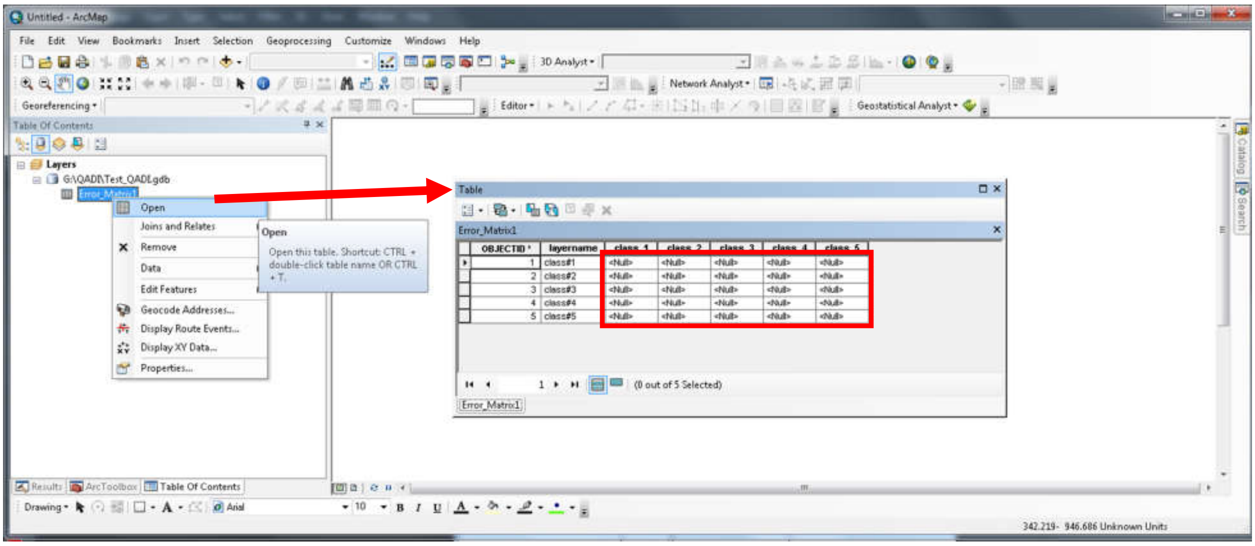

Now start editing in "Editor" icon on toolbar. That will let you manipulate cells values and insert new entries to the cells.

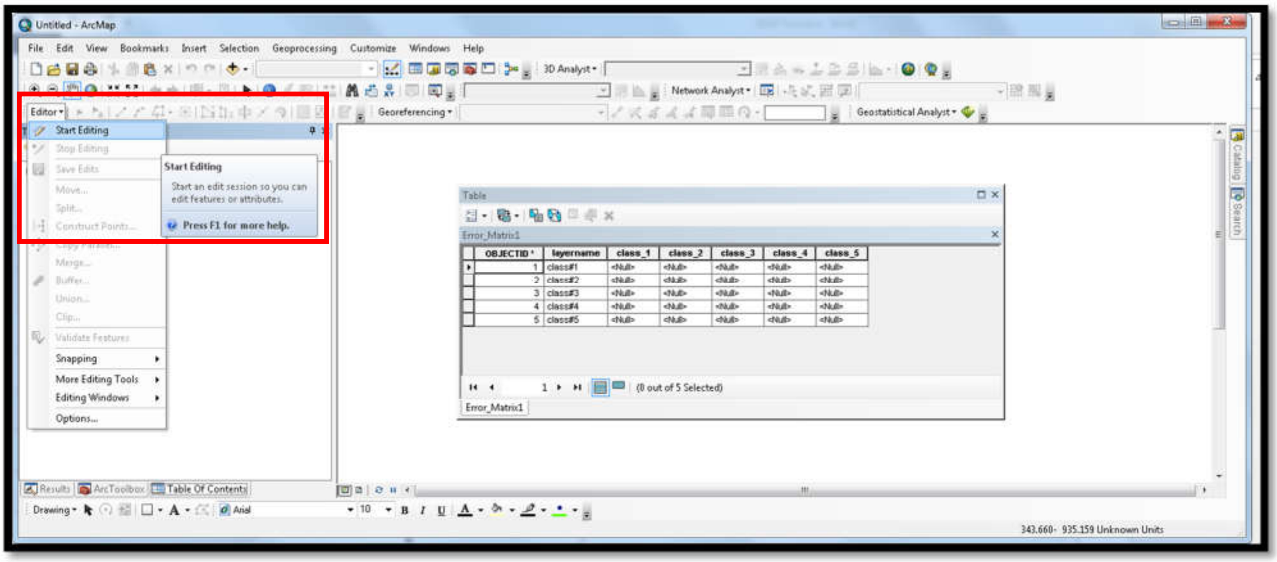

Start typing and fill the table as the error matrix is. Do it accurately and be careful to make no mistake. When done, stop editing and save the changes you made. Now it is ready to run Step 2.

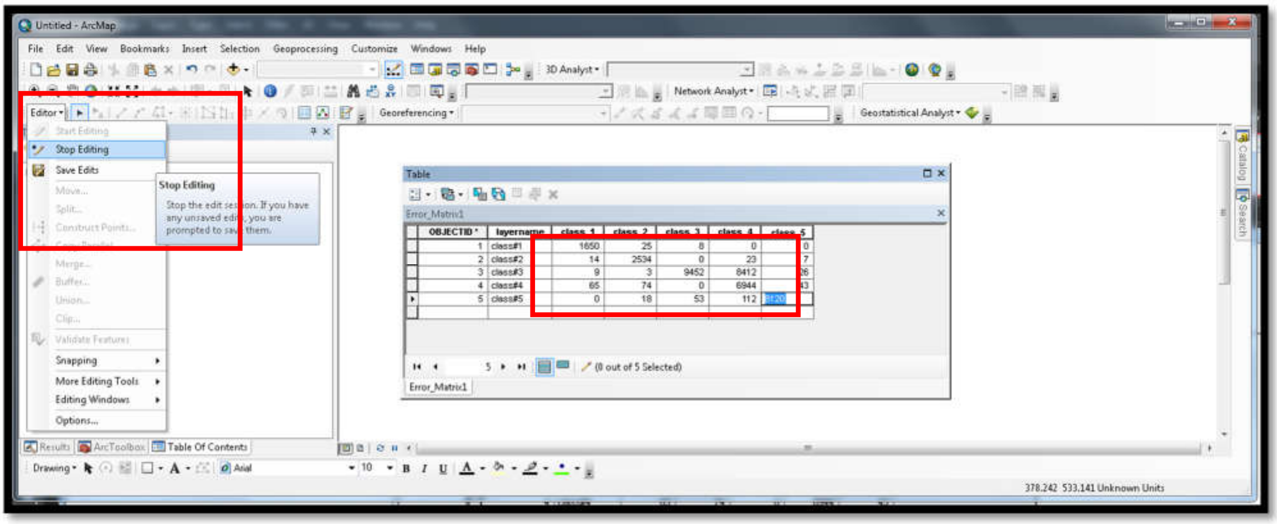

III. Running QADI toolbox in ArcMap (Step 2)

Following steps mentioned above, helps users to import their error-matrixes to ArcMap. From now on, the main function will be applied to the matrix and it will extract QADI value and its graph to do an assessment on accuracy of classification. Let's return to ArcToolbox and run second step of script. A dialogue box will appear and asks for the table you filled it bnd efore. Also The matrix rank (number of classes) and a path to save results in it. The graph name is "QADI_Graph_1" as default but it could be modified.

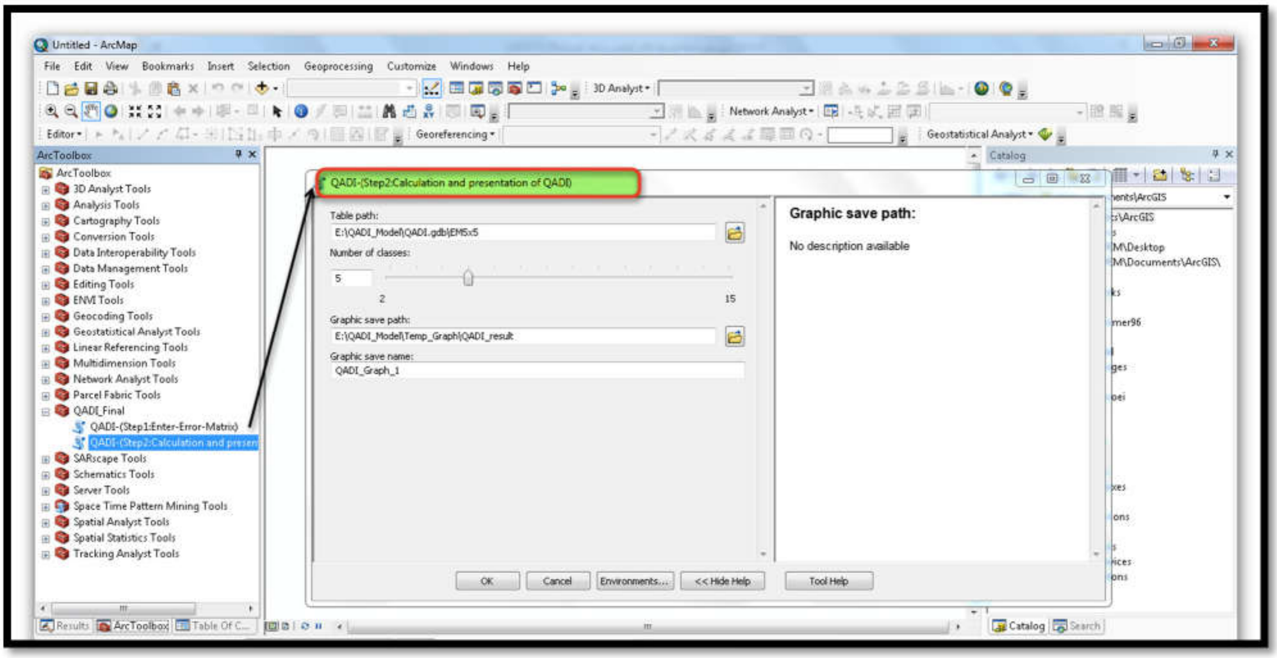

Pressing "Ok" will starts the script and pup up a graph that contains a copy of error matrix, QADI value, QADI point and other indices like Kappa and Overall accuracy. The final step is saving the graph in user defined location and needs to close the current graph. User will find the result in .PNG and .PDF format in specified path.

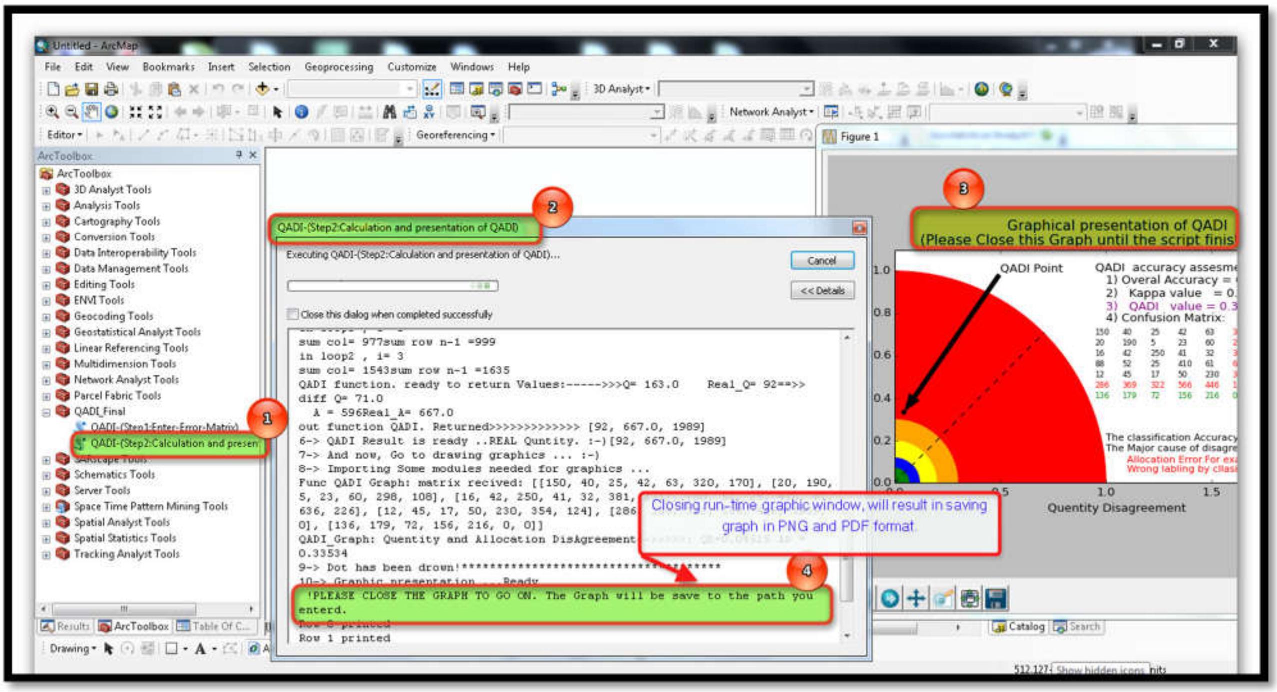

Both of saved graphs have acceptable quality to print or publish online. Here is a sample graph.

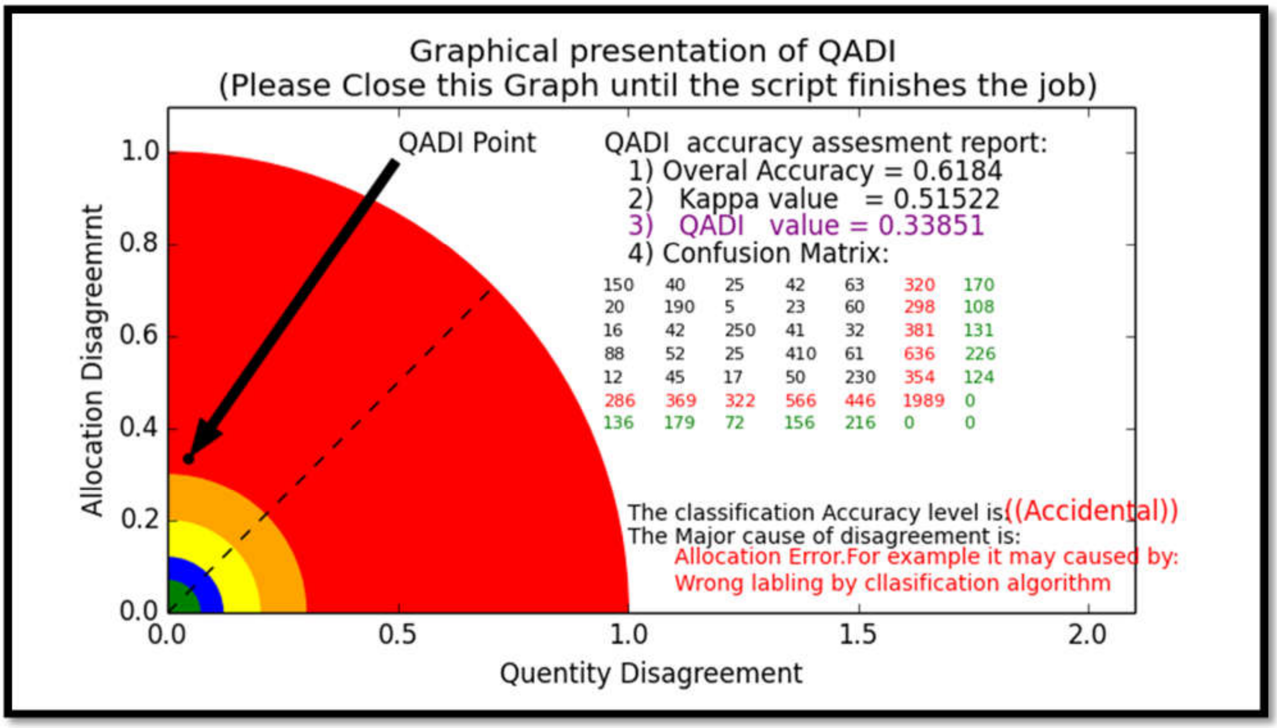

Note: The reviewers wanted us to change the color scheme of graph in manuscript. If you faced some differences between graphs in article and in your own graphs, there will be ok because you can attend to the numeric values, boundaries and axes labeling in graphs. And calibration table.

For any other question about QADI toolbox and its updates please feel free to contact us at:

darabisadra@gmail.com

feizizadeh@tabrizu.ac.ir

Best regards.
